# Tensile Property of 7075 Aluminum Alloy with Strengthening Layer by Laser Remelting-Cladding Treatment

**DOI:** 10.3390/mi14112017

**Published:** 2023-10-30

**Authors:** Qi Sui, Ning Hu, Yingrui Su, Yan Wang, Xiaolei Song

**Affiliations:** Key Laboratory of Advanced Structural Materials, Ministry of Education, School of Materials Science and Engineering, Changchun University of Technology, Changchun 130012, China; hning042800@163.com (N.H.); suyingrui0980@163.com (Y.S.); songxiaolei@ccut.edu.cn (X.S.)

**Keywords:** 7075 aluminum alloy, laser remelting-cladding, strengthening layer

## Abstract

The Ni60-SiC-CeO_2_ strengthening layer with deep remelting pools was constructed on the surface of 7075 aluminum alloy using the laser remelting-cladding processing method, and a soft and hard interphase was prepared on the matrix by the interval of laser remelting, which was inspired by soft–hard interphase structure with excellent crack inhibition performance from the natural world. The microstructure and microhardness of the remelting region and the remelting-cladding region of the strengthening layer were studied. The tensile characteristics of two distinct strengthening layers were investigated in the laboratory. The results showed that the grain size of remelting pools is finer, and the microhardness is higher than that of the matrix, which makes crack propagation more difficult. In addition, the results show that the strengthening layer has compact and flawless microstructure and has been metallurgically integrated with the matrix, and the microhardness of the regions treated by laser cladding and laser remelting-cladding has been improved obviously. Toughness has improved, as has the problem of toughness reduction after cladding ceramic particles. The sample’s strength is also significantly greater than that of the untreated sample.

## 1. Introduction

Aluminum alloy has a low density, high specific strength, thermal conductivity, good castability, and processability [1,2], making it a popular light metal. Due to its excellent comprehensive properties, aluminum alloy soon entered the fields of aerospace, automotive industry, transportation, and other fields [3,4]. The 7075 aluminum alloy offers a high specific strength and thermal treatability when compared to other aluminum alloy series [5,6]. However, aluminum alloys have poor wear resistance and mechanical properties compared to traditional steel parts, which limits their wider use [7,8]. In order to improve the mechanical properties of aluminum alloy, surface modifications are developed, including thermal spraying [9], vapor deposition [10], surface carburizing [11] and nitriding [12], micro-arc oxidation [13] and laser technology [14,15], and so on. Among them, the laser cladding method has attracted much attention in recent years [16,17].

Laser cladding method was widely utilized for modifying and repairing metal surfaces because the laser surface melting process held advantages over conventional surface processes, including good fusion, low dilution, minimum distortion [18,19]. Li et al. [20] found that the Ti/TiBCN coating produced by laser cladding on 7075 aluminum alloy had high hardness, good corrosion, and wear properties. Wang et al. [19] studied the microstructure and precipitation behavior of laser cladding 7075 aluminum alloy, and the results showed that the strength of the cladding layer was lower than that of the matrix, mainly because the quantity density of fine precipitates in the cladding layer was lower than that of the matrix. Therefore, direct laser cladding of 7075 aluminum alloy may make its strength low, but the addition of rare earth oxides can improve this situation. Wang et al. [21] prepared Ni60 cladding layer with rare earth oxides on the surface of 6063 aluminum alloy by laser cladding. It was found that the samples with rare earth oxide cladding layer had defects such as porosity and inhomogeneity reduced, grains were refined, dendrite structure was dense, and the hardness of the cladding layer surface was improved. Yanan et al. [22] found that CeO_2_ had little effect on the phase composition of the coating, and the addition of an appropriate amount of CeO_2_ would refine the microstructure of the coating, which could eliminate “herringbone” cracks and improve the microhardness and wear resistance of the coating. Liang et al. [23] found that the addition of rare earth compounds could improve the fluidity of the molten pool during the solidification process of the cladding layer, purify the molten pool, and most of the rare earth compounds were segregated at the grain boundary and phase boundary, which strengthened the grain boundary and phase boundary of the cladding layer.

However, while laser cladding technology can improve the surface hardness and wear resistance of the matrix, due to the large difference in the coefficients of thermal expansion of metals and ceramics, high residual stresses may occur at the interface during rapid solidification after laser processing [24,25]. These residual strains might cause the coating to delaminate from the matrix and shed [26]. Especially when the service surface of the cladding layer of the part shows geometric discontinuity (usually from service injuries or bolt holes, keyways, etc.), the stress strain concentration at the root of the cut at the joint is very easy to germinate cracks in this plane and the instability of cracks prolong, thereby aggravating the risk of detachment between the cladding layer and the matrix. Biomimetic, as a science that imitates the principles of biological systems to build useful component technology systems, provides us with effective ideas to improve the crack arrest capability [27,28]. In nature, for example, dragonfly wings, locust wings, and shells exhibit excellent high strength and crack arrest performance [29,30]. Zhao and Yuan et al. found that the soft–hard structure had a positive effect on the wear resistance and tensile properties of aluminum alloys [31,32].

Therefore, in this study, in order to prevent the stress concentration at the interface between the cladding layer and the matrix from causing the cladding layer to crack or even fall off, a deeper transition structure is needed as the connection between the matrix and the cladding layer. The laser remelting is applied before the laser cladding to prepare deep remelting pool to form a soft–hard interphase structure in the binding region; as a whole, it looks like a “pinning” structure layer. This structure can not only solve the problem of the cladding layer cracking, but it can also meet the requirements of not reducing toughness but also increasing its strength in some cases. Using Ni-based powder, SiC ceramic particles, and rare earth oxide CeO_2_ mixed powder as the cladding powder, the “pinning” structure layer was formed on the surface of the matrix by laser remelting and cladding method. The effect of deep remelting pools on the strengthening layer and mechanical properties of 7075 aluminum alloy was further explored, and its mechanism was summarized and analyzed.

## 2. Experimental

### 2.1. Experimental Materials 

**Figure 1:**
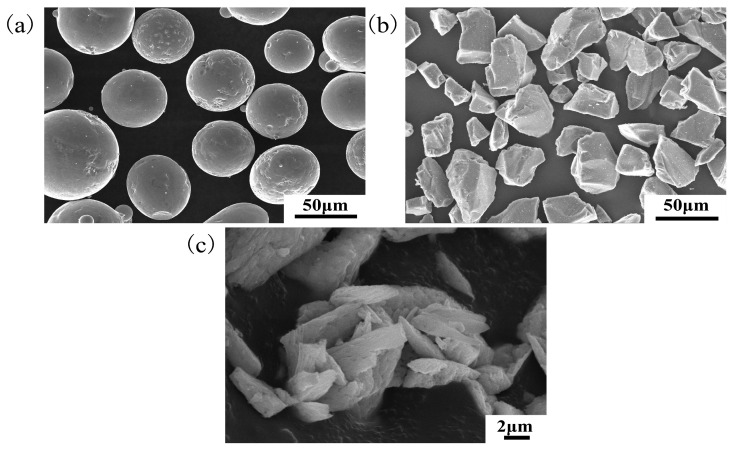
Powder SEM characterization: (**a**) Ni60; (**b**) SiC; (**c**) CeO_2_.

**Figure 2:**
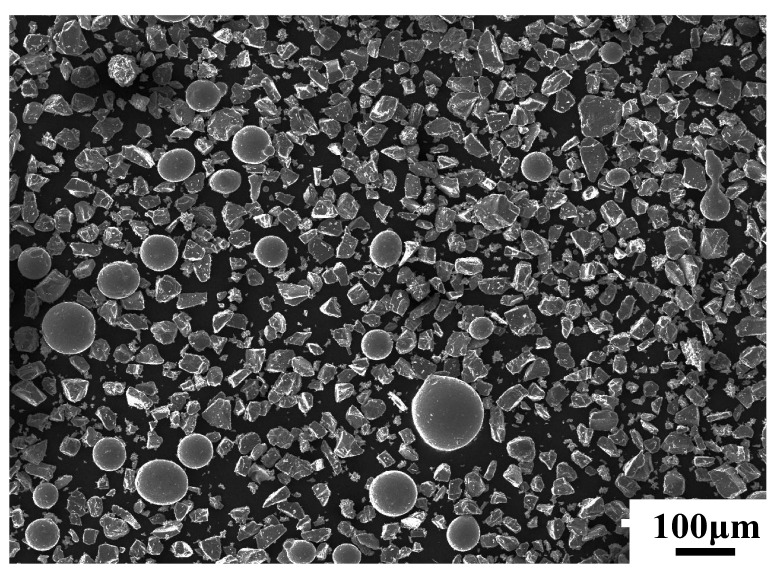
SEM of Ni60-SiC-CeO_2_ mixed powder.

In this study, the materials used were 7075 aluminum alloy, and Ni60-SiC-CeO_2_ powder for the cladding coating. Ni_60_ self-fusible powders (average particle size of 66 μm) with the remelting point of 960 °C to 1040 °C was used. SiC is irregular and granular, its specification length is between 20 and 50 μm, and CeO_2_ is long and flake, its specification length is between 2 and 9 μm. Figure 1 shows the scanning electronic photos of SiC, CeO_2_, and Ni60 powders. In order to ensure the mixing uniformity of different powders, the three powder materials were mixed by the planetary ball mill (QM-3SP2-1). The ratio of ball and material is 2:1, the mixing time is 2 h, and the speed is 150 r/min. Figure 2 shows the scanning electronic photo of Ni60-SiC-CeO_2_ mixed powder. The mass of Ni60-SiC-CeO_2_ powder was 82 wt.%, 15 wt.%, and 3 wt.%, respectively. The chemical composition of 7075 aluminum alloy is shown in Table 1.

### 2.2. Sample Preparation

**Figure 3:**
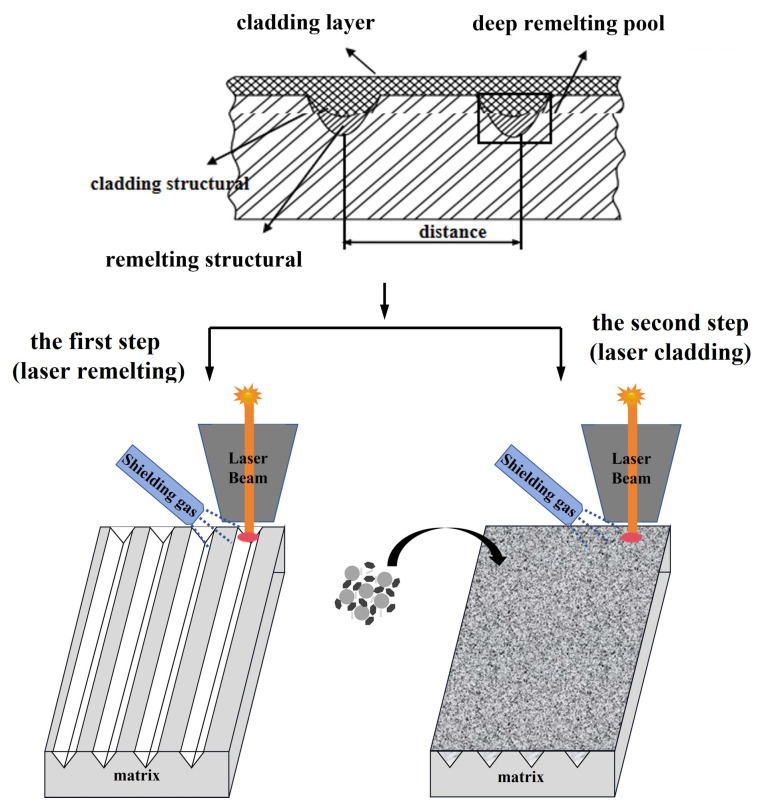
Sample preparation diagram.

The laser facility used here was an Nd: YAG laser in a pulse wave mode, operating at 1064 nm, overall power 16 KW with a Gaussian distribution of the energy in the beam. First of all, before laser remelting processing, the aluminum alloy surface is polished with coarse abrasive paper to increase the roughness, so as to increase the laser absorption of the aluminum alloy matrix. Through the movement of the worktable, the high-energy-density laser directly acts on the surface of the matrix under the air medium, so that the matrix rapidly melts and solidifies, forming a strip deep remelting pool and performance different from that of the matrix. The distance between the center lines of adjacent strip deep remelting pools is 2 mm. Then, the cermet particles mixed in liquid sodium silicate are coated on the matrix surface and remelted surface of the sample, and the coated powder is processed by secondary laser to form a strengthening layer. The cladding layer and the deep remelting pools form a “pinning” structure strengthening layer, as shown in Figure 3. The experiment was carried out at room temperature and in an argon atmosphere. By changing laser parameters, a laser with different input energy densities can be obtained. The corresponding laser processing parameters are shown in Table 2. In addition, the samples treated by laser cladding were compared with those treated by laser remelting and cladding.

**Figure 4:**
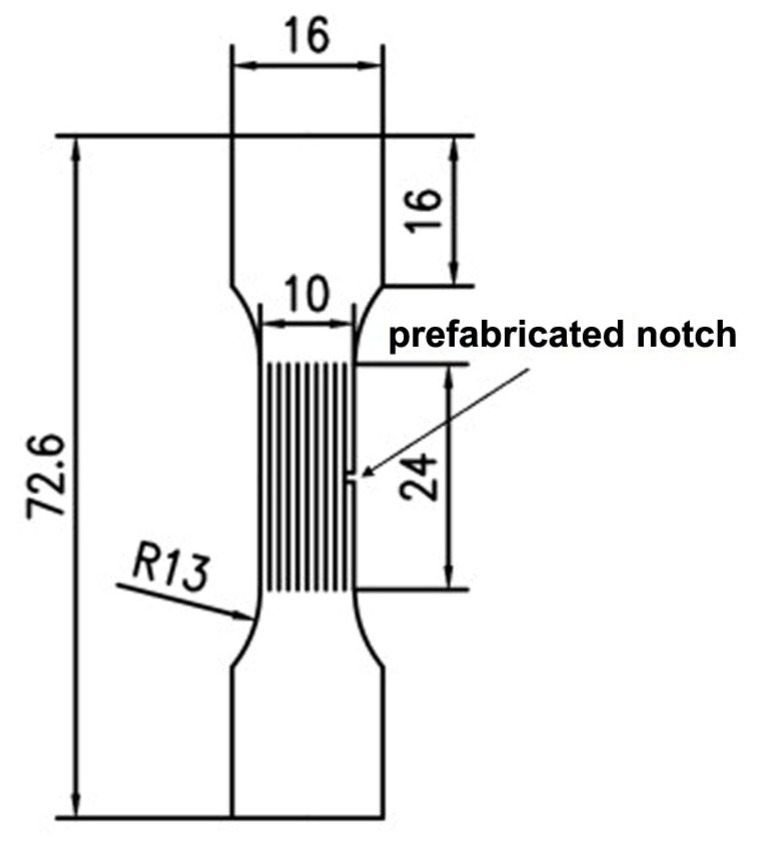
Size of tensile samples.

The tensile sample was cut into the dog bone shape, with the gauge length of 24 mm, a width of 10 mm, and a thickness of 3 mm (as shown in Figure 4). In order to observe in the process of stretching, laser remelting-cladding, and laser cladding to resistance the role of tensile crack, before the start of the tensile test, a preparatory notch, 1 mm in depth and 1 mm in width, was performed at the center of the samples’ neck to simulate an opening crack on the surface of a member (the most dangerous situation) [33]. The tensile test adopts mechanical test, controlled by servo-controlled hydraulic test system, and the strain rate is 3.5 × 10^−4^ s^−1^ at room temperature. Five repeated tests were performed on the samples for the average value.

### 2.3. Performance Characterization

Before the tensile experiment, in order to eliminate the influence of the oxide film and irregular processing marks on the surface of the sample after laser cladding, the samples were mechanically polished with progressively refined silicon carbide impregnated sandpaper. For surface contaminants, ultrasonic cleaning in alcohol is performed.

The macrostructure morphology, phase composition, and microhardness of the samples were investigated. The tensile properties were tested. Subsequently to laser processing, a cross section was cut parallel to the laser direction, where the width and depth of the remelting pool were observed under an optical microscope (Zeiss, Gemini Supra 40, Oberkochen, Germany). The microstructure and fracture morphology of tensile samples were studied by scanning electron microscopy. Molten phase structures were determined by X-ray diffraction (Rigaku D/max 2500, Tokyo, Japan) equipped with Cu Ka radiation at an operating voltage of 40 kV, a current of 40 mA, and a scanning speed of 2°/min. Moreover, the microhardness of remelting pool surface and cross section was measured at a loading of 25 g with a holding time of 15 s utilizing Vickers microhardness test.

## 3. Results and Discussion

### 3.1. Microstructure 

**Figure 5:**
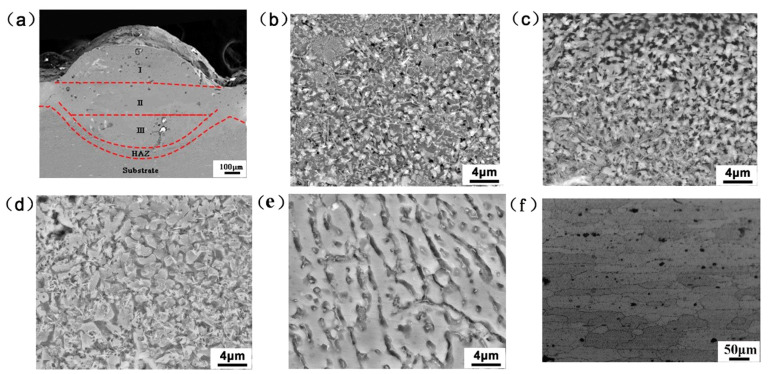
SEM microstructure of different regions of deep remelting pool: (**a**) cross-section morphology; (**b**) surface (region I); (**c**) middle (region II); (**d**) bottom (region III); (**e**) HAZ; (**f**) matrix.

The cross-section morphology of the deep remelting pool formed by the laser remelting-cladding with Ni60-SiC-CeO_2_ alloy powder is shown in Figure 5a, and the microstructure of the treated region is shown in Figure 5b–e. Figure 5a shows the cross-sectional morphology of the deep remelting region of the strengthened layer produced by the laser. The surface of the sample after laser processing is melted in a local region due to the energy of the laser, and then rapidly solidified. Since there are different temperature gradients along the direction of laser processing, the phenomenon of delamination between the remelting region and the matrix will be caused. The top of the melted zone is wider than the bottom after processing. Bottom profiles are considered inverted parabolic. It can be seen that the grains inside the remelting pool gradually increased in size with the increase in depth. As seen in Figure 5b, the microstructure of the deep remelting pool at the region I was mainly dense equiaxed crystals and dendrites, and the second phase was uniformly dispersed in the dendrite gaps. As shown in Figure 5c, the main morphology of the grains in the middle of the deep remelting pool (region II) was relatively dense equiaxed grains, which was due to the fact that the heat in the middle of the remelting pool was not easy to dissipate and the degree of undercooling was large. The microstructure at the bottom of the deep remelting pool (region III) is depicted in Figure 5d, and it is primarily made up of equiaxed grains with high grain sizes, with the distribution of the second phase being reduced. The microstructure of laser cladding HAZ is shown in Figure 5e, which is mainly composed of relatively coarse dendrites and gradually transits from dendrites to matrix structure, mainly due to the combination of remelting pool and matrix, and the laser energy is transferred to the matrix, causing local 7075 aluminum alloy matrix damage, showing relatively coarse Al-rich phase. Figure 5f shows the grain structure in the initial state of the matrix, and the grain composition is a strip with consistent direction. It can be observed that the grain morphology and size change obviously from the molten pool to the matrix. In the molten pool, the refined structure is mainly caused by the temperature gradient (G) and solidification rate (R), especially the parameter G/R [34]. In this region, G is very small, and R is very high, i.e., G/R is very low. It tends to form fine equiaxed dendrites, as shown in Figure 5b. In the heat affected zone, the dendrite growth is mainly affected by the direction of heat flow due to the heat transfer of the matrix. Since the direction of heat flow is perpendicular to the interface between the heat affected zone and the matrix, and the growth direction of dendrites is opposite, the dendrites in the heat affected zone are perpendicular to the interface, as shown in Figure 5e.

**Figure 6:**
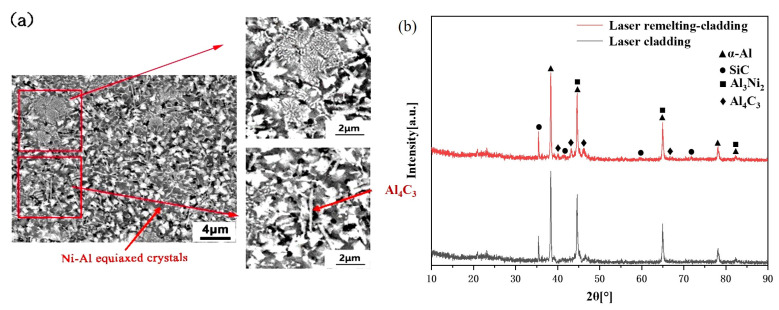
XRD of 7075 aluminum alloy surface by laser remelting-cladding. (**a**) the microstructure of remelting-cladding layer; (**b**) the XRD patterns of surfaces treated by laser cladding and laser remelting-cladding.

Figure 6 shows the XRD pattern of the laser remelting-cladding 7075 aluminum alloy surface. The XRD patterns showed that in addition to SiC, α-Al, and other phases in the composite coating and matrix, there were also compounds formed by Ni, SiC, and Al matrix on the surface of the remelting-cladding layer. From Figure 6a, it can be seen that a large number of light gray Ni-Al equiaxed crystals are uniformly distributed in the cladding layer. The Ni-Al equiaxed crystals were mainly Al_3_Ni_2_ generated by the chemical reaction of Ni and Al, and there are irregularly arranged needle-like carbides around this phase. The carbides were mainly Al_4_C_3_ formed by the eutectic reaction of SiC and Al. With the observation to the depth of the molten pool, the grains gradually coarsed, the Ni-Al phase and carbide gradually increased, and needle-like carbides overlapped each other to form a network structure surrounding Ni-Al phase locally. A large number of coarse Al-rich equiaxed grains can be seen at the bottom of the deep-remelting unit. The change of the microstructure in each section of the deep remelting region mainly depended on the ratio of the temperature gradient and the solidification rate during the solidification process of the molten pool. Since the surface layer had good diffusivity in contact with air, the degree of supercooling was increased, thereby increasing the nucleation rate of the structure, and making the grains of the surface layer structure relatively fine. In the middle of the molten pool, the heat was not easily dissipated, which reduced the degree of subcooling, so that Ni, SiC, and molten Al react sufficiently, forming the structure in Figure 5c. At the bottom of the deep remelting region, due to the residual heat transferred to the bottom layer during the high-energy density laser remelting and surface laser cladding processes, the degree of subcooling was reduced, the crystal growth time was prolonged, and the Al remelting in the structure occurred. The coarse Al-rich equiaxed crystals shown in the SEM images were formed. By comparing the XRD patterns of surfaces treated by laser cladding and laser remelting-cladding in Figure 6b, it is found that the main phase composition is almost the same, containing SiC, α-Al, Al_3_Ni_2_, and Al_4_C_3_. However, the higher peak strength of the hard phases of SiC and Al_4_C_3_ was detected on the surface of the laser remelting-cladding, which is speculated that the main reason is the first step treatment with high-energy beam remelting refined the microstructure of the matrix. The dense microstructure leads to improved interatomic bonding force. Compared with laser cladding on aluminum alloy matrix, the solution fluidity in the dense remelting region decreased and the viscosity increased during laser cladding in the remelting region. It is difficult for C and Si with large atomic radius to diffuse deep into the remelting region and stay in the near surface, so that the density of SiC and Al_4_C_3_ in the near surface increased, and higher peak strength of them was detected on the surface of the laser remelting-cladding. It also caused an increase in the microhardness of the region.

### 3.2. Microhardness 

**Figure 7:**
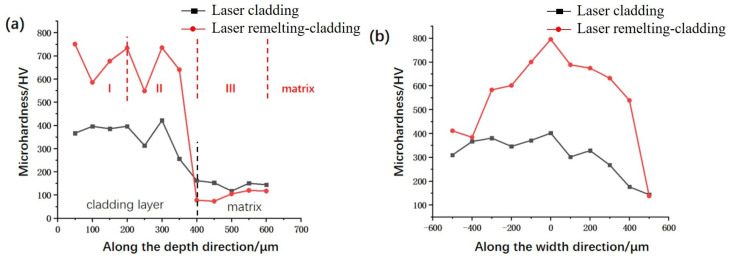
Different processing methods and different directions of microhardness. (**a**) along the depth direction with I, II, III and matrix regions; (**b**) along the width direction.

The microhardness profiles along the depth and the width directions in the cross sections of the remelting-cladding region and the cladding region are shown in Figure 7a,b respectively. Compared with the cladding region, the microhardness of the remelting-cladding region was significantly improved, mainly because of the presence of more hard phases near the surface. The microhardness curves along the depth direction of both samples showed a decreasing trend from the surface to the matrix. The main reason was that the alloying elements in the alloy powder were melted and diffused into the molten pool by heating, which increased the microhardness. The diffusion of alloying elements decreased with the increase of depth, and the Al, Mg, Si, and other elements in the matrix were heated to melt and diffuse into the molten pool [21]. It can be seen from Figure 7a that the distribution trends of the microhardness along the depth direction and the width direction are almost the same under the cladding region treated on the aluminum alloy matrix and on the remelting matrix. From the microhardness along the width direction in Figure 7b, it can be seen that the microhardness of in the middle is higher, and the microhardness along the width direction shows a decreasing trend. It is mainly because the laser energy absorbed by the middle region is higher, which means more alloy powders can be melted.

### 3.3. Tensile Results

**Figure 8:**
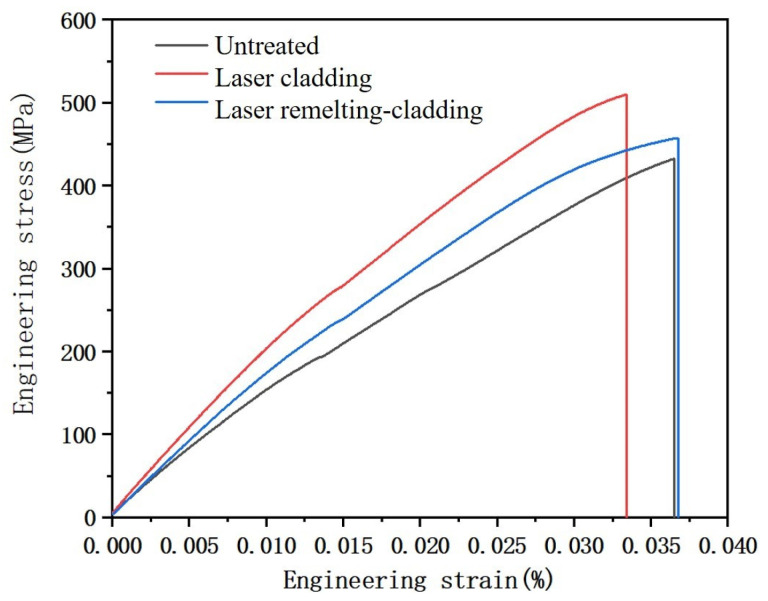
Tensile stress–strain curves of different samples.

Typical tensile stress–strain curves of different samples are shown in Figure 8, and Table 3 lists the average values of the ultimate tensile strength (UTS) and the elongation (EL), which were calculated from the stress–strain curves. It showed that samples with the biomimetic strengthening layer (laser remelting-cladding sample) and with the cladding strengthening layer (laser cladding sample) presented different tensile results. The EL and the UTS of the laser remelting-cladding sample were 0.0367% and 456.73 Mpa, respectively. The EL and the UTS of the laser cladding sample were 0.0334% and 509.35 Mpa, respectively. The EL and the UTS of the untreated sample were 0.0365% and 430.20 Mpa, respectively. Obviously, the UTS of the samples treated by laser surface strengthening technology was obviously improved, and the UTS of the laser cladding sample was the highest, followed by the laser remelting-cladding sample and the untreated sample. Compared with the untreated sample, the strength of the laser cladding sample was significantly improved, but the elongation was reduced, because the addition of ceramic particles during cladding consumes more energy during stretching. It increased the tensile strength when stretched, but it was also accompanied by a problem of reduced toughness. Corresponding to this, the laser remelting-cladding sample showed different results from the laser cladding sample. The tensile strength was lower than that of the laser cladding sample, but it was significantly higher than that of the untreated sample. The toughness was also roughly the same as the original untreated test, indicating that the sample after laser remelting-cladding treatment can correspondingly increase the strength of the sample without reducing the toughness. In comparison, the laser remelting-cladding sample exhibited superior tensile properties, because the laser remelting-cladding sample undergoes two laser processes during the processing, resulting in a higher degree of grain refinement and higher plasticity. so that the tensile effect of the sample was better.

### 3.4. Analysis of Fracture Morphology

**Figure 9:**
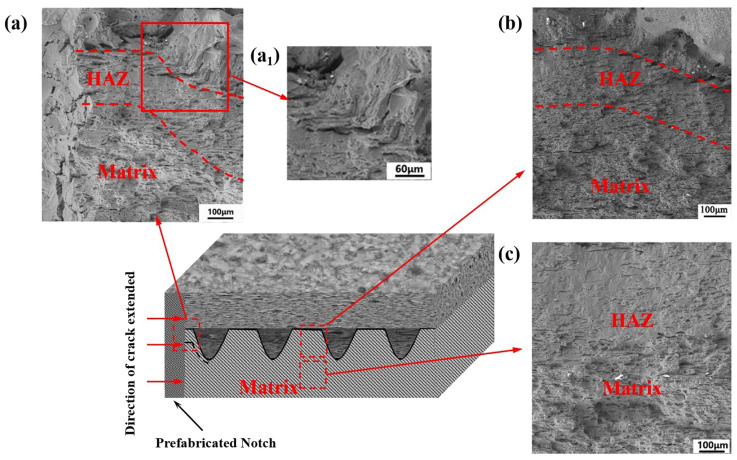
Tensile fracture morphology of 7075 alloy surface by laser remelting-cladding: (**a**) near the notch with remelting-cladding layer; (**a_1_**) enlarged view of deep remelting pool near the notch region; (**b**) deep remelting pool located near the middle region; (**c**) the heat affected region and the matrix region near the middle of the remelting pool.

**Figure 10:**
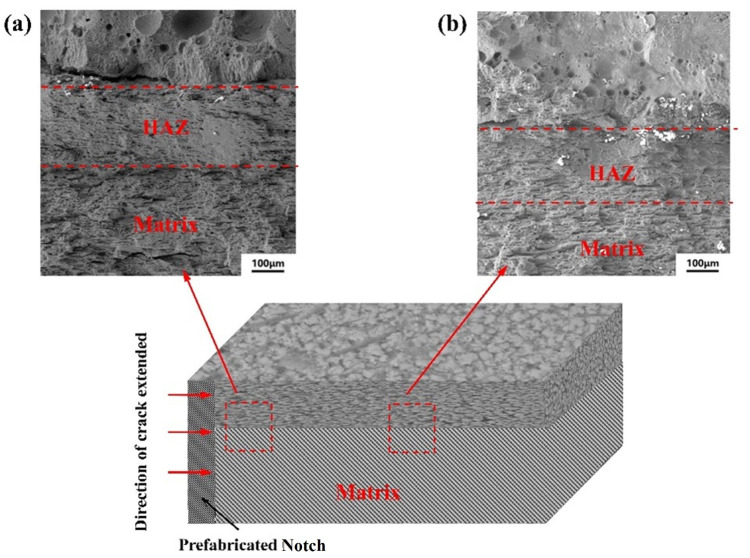
Tensile fracture morphology of 7075 alloy surface by laser cladding: (**a**) the layer is near the incision; (**b**) the layer is near the middle.

Figure 9 and Figure 10 show the fracture morphology of the cladding and remelting-cladding samples after tension. When the sample is stretched, the crack propagation direction is as shown in the figure. In Figure 9a, it can be clearly observed that the cracks near the remelting-cladding layer have a deflection phenomenon after straight line propagation. In Figure 9a_1_, it can be clearly seen that the cracks are blocked by the deep remelting pool when passing through the deep remelting pool and do not continue to move forward, with large cleavage steps. It shows that more energy is required to continue to expand along the crack growth direction. In Figure 10a, it is obvious that a relatively wide crack appears between the strengthened layer and the matrix, and the crack extends directly along the extension direction without deflection.

It can be seen from the figure that, due to the existence of the deep remelting pool, three different fracture regions are shown, which are divided by the dotted line in Figure 9a: the strengthening layer region, the heat affected region, and the matrix region. Due to the existence of the deep remelting pool, the crack growth is blocked when the crack grows near the element body, showing the appearance marked by the dotted line in the figure, while the crack in Figure 10a is restrained and continues along the crack growth direction. As can be seen in Figure 9b and Figure 10b, the tensile morphology of the specimen near the middle of the notch also presents different shapes. When the crack propagates near the deep remelting unit near the middle region, it is still blocked by the deep remelting pool, and the crack is small and short. The crack is near the notch of the cladding layer, and it extends along the junction between the matrix and the strengthening layer. Because the crack consumes a lot of energy at the notch, it decreases in the middle. Figure 9c is divided into upper and lower parts. It can be clearly seen that in the matrix part, the fracture surface is mainly composed of tear edges and dimples. In the heat affected region, cleavage steps and river patterns can be clearly seen, indicating that the fracture modes of the upper and lower parts of the sample are different. When observing the tensile morphology, it is observed that the complete brittle fracture surface of partial ceramic falling off may be due to the formation of the hard phase during the processing. When the crack extends to the hard phase, the energy is not enough to pass through the hard phase and deflect from nearby, resulting in falling off.

### 3.5. Fracture Mechanism Analysis

**Figure 11:**
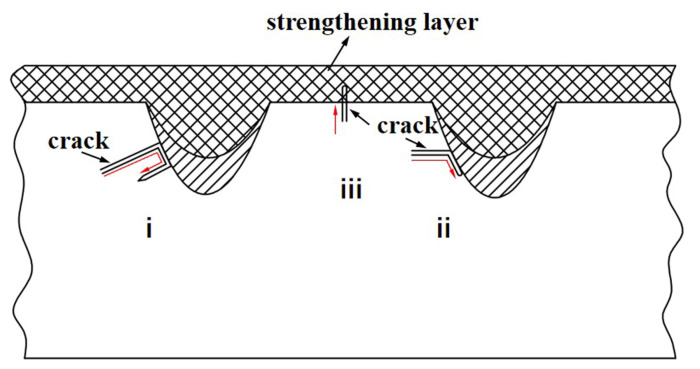
The mechanism by which cracks with i, ii and iii propagation types propagate from the matrix to the remelting-cladding layer.

Figure 11 is a schematic of the crack propagation mechanism. It can be seen from the figure that there are three crack propagation mechanisms: the first crack is deflected to the matrix again after reaching the strengthening layer (type i); the second is that the crack continues to propagate in the direction of propagation (type ii); and the third is that the crack passes through the low strength region where the bonding region of strengthening layer and the matrix (type iii). When the crack extends from the matrix to the strengthening layer, due to the inhibition of the expansion of the strengthening layer, it slowly propagates along the bottom of the strengthening layer. Before the driving force is completely consumed, the crack changes direction and enters the matrix with better expansion, showing the first type of crack expansion mechanism. In the second type, there are two forms of crack propagation. One form is to reach the strengthening layer from the matrix along the expansion route, and the hardness of the strengthening layer produces resistance to the continuous expansion of the crack, which inhibits the further expansion of the crack; the other is that the crack propagates in the strengthening layer. The other is the crack generated at the interface between the strengthening layer and the matrix. Most of the cracks propagate along the bottom of the strengthening layer. The propagation path of the crack varies with the difference in the morphology of the strengthening layer between the two samples. When the parabolic biomimetic strengthening layer with units expands, its expansion path is longer than that of the biomimetic strengthening layer without units. A third propagation mechanism occurs in the laser-treated overlap region, where the hardness may be reduced due to tempered effect [35], where cracks initiate and propagate.

For the tensile morphology with a biomimetic strengthening layer, it can be seen from the crack propagation path that when the crack extends along the matrix to the strengthening layer, the continuous propagation of the crack along the original direction is hindered. Under the conditions of rapid laser heating and cooling, the microstructure after laser remelting-cladding treatment is smaller than that of the matrix (Figure 5). Grain refinement can improve the hardness and fracture toughness of the material. The grain refinement increases the grain boundary, and the crack is difficult to propagate along the fine strengthened grain. Compared with linear growth, grain refinement prolongs the crack growth path and induces crack migration. More frequent crack deflection increases the energy absorbed during deformation, which helps to reduce crack growth and makes it difficult for the crack to maintain the direction of bionic element growth. At the same time, compared with the structure of laser cladding cermet powder, the hardness of the structure after laser remelting treatment is relatively low. The alternative toughening structure, which has two hardness, helps to increase tensile strength without sacrificing good toughness.

## 4. Conclusions

In this paper, Ni-SiC-CeO_2_ ceramic alloy powder was used on the surface of 7075 aluminum alloy by laser remelting-cladding technology to prepare a “pinning”-like structure. The effects of two different processing methods on the microhardness, microstructure, and tensile properties of 7075 aluminum alloy were investigated, and the tensile mechanism was analyzed. The conclusions were summarized as follows:

1. Compared with the untreated samples, the microstructure of the treated samples was more compact. In addition, the microhardness of treated samples was significantly increased markedly improved.
2. Compared with the samples with cladding layer, the strength of samples with the remelting-cladding layer was reduced, but its toughness was significantly improved, which not only meet the requirements of toughness but also could increase the strength of materials. After stretching, the fracture surface of the two samples showed a small number of dimples and tearing edges, which showed quasi-cleavage fracture.
3. The stretching mechanism was studied. The grains of the remelting-cladding layer with deep remelting pools were more refined and the structure was denser, which hindered the initiation of cracks and prolonged the crack path.

## Figures and Tables

**Table 1:** Chemical composition of 7075 Al alloy (wt.%).

Si	Fe	Cu	Mn	Mg	Cr	Zn	Ti	Al	Others
0.06	0.16	1.51	0.04	2.63	0.22	5.53	0.03	Bal	<0.15

**Table 2:** Laser processing parameters of different processing methods.

	Current A	Pulse Duration ms	Frequency Hz	Scanning Speed mm/min	Defocus Value Mm
Laser cladding	120	5	5	30	5
Laser remelting	95	5	10	30	0

**Table 3:** Tensile property test results.

Sample	EI, %	UTS, MPa
Untreated	0.0365	430.20
Laser Cladding	0.0334	509.35
Laser Remelting-Cladding	0.0367	456.73

## Data Availability

No new data were created or analyzed in this study. Data sharing is not applicable to this article.
